# Protein Kinase PKN1 Represses Wnt/β-Catenin Signaling in Human Melanoma Cells*[Fn FN2]

**DOI:** 10.1074/jbc.M113.500314

**Published:** 2013-10-10

**Authors:** Richard G. James, Katherine A. Bosch, Rima M. Kulikauskas, Peitzu T. Yang, Nick C. Robin, Rachel A. Toroni, Travis L. Biechele, Jason D. Berndt, Priska D. von Haller, Jimmy K. Eng, Alejandro Wolf-Yadlin, Andy J. Chien, Randall T. Moon

**Affiliations:** From the Departments of ‡ Pharmacology and; ‡‡ Genome Sciences,; § Institute for Stem Cell and Regenerative Medicine,; ** Howard Hughes Medical Institute,; ‖ Department of Medicine, Division of Dermatology, and; §§ Proteomics Resource, University of Washington and; ¶ University of Washington School of Medicine, Seattle, Washington 98109

**Keywords:** Apoptosis, β-Catenin, Melanoma, Phosphorylation, Wnt Signaling, GSK3-β, PKN1

## Abstract

Advances in phosphoproteomics have made it possible to monitor changes in protein phosphorylation that occur at different steps in signal transduction and have aided the identification of new pathway components. In the present study, we applied this technology to advance our understanding of the responses of melanoma cells to signaling initiated by the secreted ligand WNT3A. We started by comparing the phosphopeptide patterns of cells treated with WNT3A for different periods of time. Next, we integrated these data sets with the results from a siRNA screen that targeted protein kinases. This integration of siRNA screening and proteomics enabled us to identify four kinases that exhibit altered phosphorylation in response to WNT3A and that regulate a luciferase reporter of β-catenin-responsive transcription (β-catenin-activated reporter). We focused on one of these kinases, an atypical PKC kinase, protein kinase N1 (PKN1). Reducing the levels of PKN1 with siRNAs significantly enhances activation of β-catenin-activated reporter and increases apoptosis in melanoma cell lines. Using affinity purification followed by mass spectrometry, we then found that PKN1 is present in a protein complex with a WNT3A receptor, Frizzled 7, as well as with proteins that co-purify with Frizzled 7. These data establish that the protein kinase PKN1 inhibits Wnt/β-catenin signaling and sensitizes melanoma cells to cell death stimulated by WNT3A.

## Teaser

**Background:** Wnt/β-catenin signaling inhibits melanoma cell viability.

**Results:** Integration of phosphoproteomics and RNA interference screens querying the Wnt/β-catenin pathway reveals protein kinase N1 as an inhibitor of signaling.

**Conclusion:** Protein kinase N1 inhibits Wnt/β-catenin signaling and apoptosis in melanoma cells.

**Significance:** This study identifies a kinase that inhibits Wnt/β-catenin signaling, a pathway critical to melanoma cell viability.

## Introduction

The 19 members of the Wnt family of secreted glycoproteins regulate diverse intracellular signal transduction cascades in vertebrates. One Wnt pathway, herein referred to as the Wnt/β-catenin pathway, is activated by the binding of Wnt ligand to Frizzled (FZD)^5^ serpentine receptors and to low density lipoprotein receptor-related protein (LRP) co-receptors (for reviews, see Refs. 1–3). Following receptor activation, a series of events result in stabilization of nuclear and cytosolic pools of the multifunctional adapter protein β-catenin (encoded by *CTNNB1*). β-Catenin then promotes transcriptional changes that can result in modulation of differentiation and/or cell proliferation (4).

β-Catenin signaling plays diverse roles in embryonic development and in adults as well as following acute injury or chronic disease. For example, β-catenin specifies premigratory neural crest to adopt a melanocyte fate during development (5). In melanoma, a cancer generally considered to arise from melanocytes, β-catenin levels decrease during the progression of the disease (6). Higher expression of β-catenin in tumors at the time of diagnosis correlates with improved patient survival in melanoma (6–10), which is opposite of the correlation observed with colorectal cancer. Understanding the roles and mechanisms of β-catenin signaling may thus inform us about disease as well as development.

Changes in the phosphorylation of proteins play a central regulatory role in Wnt/β-catenin signaling (1–3). Specifically, in the absence of Wnt stimulus, β-catenin is constitutively degraded by the proteasome following its phosphorylation by the kinases casein kinase 1α (encoded by *CSNK1A1*) and glycogen synthase kinase 3 (GSK3) (11). Conversely, following Wnt stimulation, phosphorylation of the cytoplasmic tail of the FZD co-receptor LRP5/6 is thought to be necessary for the subsequent stabilization of CTNNB1. Upon phosphorylation of LRP5/6, the cytosolic pools of the scaffolding protein AXIN1 and its associated kinases, CSNK1A1 and GSK3, bind LRP5/6 at the cytoplasmic side of the plasma membrane (12, 13), thus allowing newly translated β-catenin to accumulate in the cytosol, translocate to the nucleus, and regulate the transcription of target genes. Therefore, screening for kinases that regulate the Wnt/β-catenin pathway in melanoma is warranted given the continual discovery of new tissue-specific points of regulation of this pathway by kinases (14, 15).

To advance the characterization of changes in protein phosphorylation upon stimulation of the Wnt/β-catenin pathway, in the present study, we defined the WNT3A-regulated phosphoproteome in a melanoma cell line. We then integrated our phosphoproteomics data with a siRNA screen targeting known and predicted kinases. This approach identified protein kinase N1 (encoded by *PKN1*) as a putative inhibitor of the Wnt/β-catenin pathway. To begin to address the mechanisms by which PKN1 might attenuate Wnt/β-catenin signaling, we demonstrated that PKN1 inhibits WNT3A-dependent phosphorylation of LRP6 and regulates its expression on the cell surface. Suggesting that PKN1 might be relevant to future therapies in melanoma, we found that depletion of PKN1 can sensitize melanoma cells to cell death initiated by the WNT3A ligand.

## MATERIALS AND METHODS

### 

#### 

##### Quantitative Phosphoproteomics

To perform stable isotope labeling with amino acids in cell culture (16) experiments, two groups of A375 malignant melanoma cells were cultured separately, one with labeled heavy amino acids and the other with light normal amino acids. After stimulation of 2.5 × 10^7^ heavy A375 cells (∼1 15-cm plate) with recombinant WNT3A (Peprotech, Rocky Hill, NJ) or CHIR-99021 (Cayman Chemical, Ann Arbor, MI) and 2.5 × 10^7^ light A375 cells with vehicle, the cells were lysed with 3 ml of 8 m urea supplemented with 1 mm Na_3_VO_4_. Cell lysates from the heavy and light cells were combined at a 1:1 protein ratio, reduced, alkylated, trypsinized, and desalted as described previously (17)

For the phosphopeptide enrichment, we adapted a previously published method from Villén and Gygi (18). Briefly, peptides were bound to a hand-packed chromatography column (strong cation exchange resin, BMSE1203, Nest Group, Southborough, MA) and washed and eluted using a P1 LKB peristaltic pump (GE Healthcare). The flow rate was maintained at 0.5 ml/min, and the following elution steps were used for the 13 8-min fractions: 0.0% elution buffer (EB; 5 mm KH_2_PO_4_, 30% acetonitrile, 350 mm KCl, pH 2.65), 2.5% EB, 5% EB, 7.5% EB, 10% EB, 12.5% EB, 15% EB, 17.5% EB, 20% EB, 22.5% EB, 25% EB, 100% EB, 100% H_2_O. The immobilized metal affinity chromatography enrichment was performed as described previously (18).

Phosphopeptides from each fraction were loaded onto a 3-cm self-packed C_18_ capillary precolumn (POROS RL2 10-μm beads; inner diameter, 100 μm; outer diameter, 360 μm). The precolumn was connected to a 20-cm self-packed C_18_ (Monitor 5 μm, Orochem) analytical capillary column (inner diameter, 50 μm; outer diameter, 360 μm) with an integrated electrospray tip (∼1-μm orifice). Online peptide separation was performed on a nano-LC system (nanoAcquity UPLC system, Waters Corp.). Peptides were eluted from the column using a 240-min gradient with solvents A (H_2_O/formic acid, 99.9:1 (v/v)) and B (acetonitrile/formic acid, 99.9:1 (v/v)) as follows: 10 min from 0 to 10% B, 180 min from 10 to 40% B, 15 min from 40 to 80% B, and 35 min with 100% A. Eluted peptides were directly electrosprayed into an LTQ Orbitrap XL mass spectrometer (Thermo Fisher Scientific, Rockford, IL). The mass spectrometer was operated in data-dependent mode in which full scans (from *m*/*z* 300 to 1500) were acquired in the Orbitrap analyzer (resolution, 60,000) followed by MS/MS analyses using collision-induced dissociation on the top 10 most intense precursor ions.

##### Data Analysis

MS/MS data files were searched using the SEQUEST (19) algorithm. Variable (phosphorylation of serine, threonine, or tyrosine, 79.8 Da; heavy arginine, 10.0 Da; heavy lysine, 6.0 Da) and static (carbamidomethylation of cysteine, 57.02 Da) modifications were used in the search. The data were further processed using the Institute for Systems Biology Trans-Proteomic Pipeline (20), and all peptides whose probability score exceeded the peptide probability score associated with a <2.5% false discovery rate were retained. Finally, the software suite XPRESS (21) was used to quantify the ratios of heavy and light peptides. The data were normalized to the sample-wide heavy to light ratio and transformed to log_2_. For all unique peptides that were sampled multiple times in our analysis, we did not consider those that had individual replicates whose normalized XPRESS ratios were greater or less than 2-fold from the mean value of all the replicates or those whose mean value for a given charge state was greater or less than 2-fold different from the mean value of another charge state.

##### Affinity Purification-Mass Spectrometry

Affinity purification was performed as described previously (17, 22). For PKN1, two independent affinity purifications were performed in A375 cells. All prey proteins that were present in both preparations and were identified by two independent peptides in one preparation were kept for further analysis. For FZD7, we analyzed all proteins containing two independent peptides. To simplify our analysis, all peptides previously demonstrated to be common contaminants using similar approaches (22) were eliminated from further analysis. Additionally, because we were analyzing two proteins that are possibly trafficked to the plasma membrane, we also eliminated proteins previously shown to localize to the endoplasmic reticulum. To identify literature interactions for PKN1 and FZD7 and to create the protein-protein interaction network for the WNT3A-dependent phosphoproteins, in-house Python scripts were used to identify literature-curated protein-protein interactions from the STRING database (23), BioGRID (24), and Human Protein Reference Database (25). The protein-protein interaction binary files and the primary mass spectrometry data were used to generate Cytoscape (26) diagrams.

##### High Throughput siRNA Screen

Screening was performed at the Quellos High Throughput Screening Facility at the University of Washington's Institute for Stem Cells and Regenerative Medicine (Seattle, WA). A library of siRNAs targeting primarily the human kinome (Ambion, Grand Island, NY) was resuspended in ribonuclease-free water. siRNA pools were screened in quadruplicate at 1.9 nm final concentration. To assess cell viability, resazurine (Sigma-Aldrich) was added (1.25 μg/ml), and the fluorescence intensity (excitation, 530 nm; emission, 580 nm) was quantified using an Envision multilabel plate reader (PerkinElmer Life Sciences). To assess luciferase activity, Steady-Glo (5 μl/well; Promega) was added, and total luminescence was quantified using an Envision multilabel plate reader (PerkinElmer Life Sciences).

##### Reagents

The reporters are lentiviral vectors containing transcription factor binding sites that respond to activation of the Wnt/β-catenin (27), and nuclear factor κB (28) signaling pathways. FZD5, FZD7, and LRP6 were all cloned by standard PCR methods (with removal of their endogenous signal sequences) into lentiviral vectors containing a CMV promoter and a puromycin resistance gene following an internal ribosomal entry site. The proteins were expressed as translational fusion products with an N-terminal muscarinic signal sequence and either an N-terminal HA tag (FZD5), an N-terminal Glue tag (FZD7; see Ref. 29), or a C-terminal Venus tag (FZD5 and LRP6).

The following primers were used for quantitative PCR (Integrated DNA Technologies): *GAPDH*: 5′-TGAAGGTCGGAGTCAACGGA, 5′-CCATTGATGACAAGCTTCCCG; *PKN1*: 5′-AAAGCAGAAGCCGAGAACAC, 5′-ACACAGCCAACTCCAGTTCC; *PKN2*: 5′-GAATCATCCCAAAAGCAGGA, 5′-CCGTCCAGGGACATTCTCTA; *PKN3*: 5′-CGATCCCGCGTCTCCGGCG, 5′-TCTGGGGGCCACTGGCTCG; and *AXIN2*: 5′-GCGATCCTGTTAATCCTTATCAC, 5′-AATTCCATCTACACTGCTGTC. The following sequences were used for siRNA experiments: Ambion PKN1-1, GCACUGUGCUUAAGCUGGAtt; Ambion PKN1-2, ACAGCGACGUGUUCUCUGAtt; Dharmacon PKN1-1, CCUCGAAGAUUUCAAGUUC; Dharmacon PKN1-2, ACAGUAAGACCAAGAUUGA; Dharmacon PKN1-3, ACAGCGACGUGUUCUCUGA; Dharmacon PKN1-4, GAGAAGCAGUUGGCCAUUG; Ambion PKN2-1, GACGAGAAGAUGUUAGUAAtt; Ambion PKN2-2, GCACCCAUUUUUCCGGCUAtt; Ambion PKN3-1, GGAACGCAUCUUCUCUAAAtt; Ambion PKN3-2, AGACCUUUGUCAUCCCACUtt; Ambion CTNNB1; GGAUGUUCACAACCGAAUUtt; Negative Control-1 (Ambion); Negative Control-1 (Dharmacon); and Negative Control-2 (Dharmacon). Murine recombinant WNT3a (Peprotech) was used in the phosphoproteomics studies and many assays for WNT3a-dependent signaling. The following compounds were used in the phosphoproteomics and viability experiments: CHIR-99021 (Cayman Chemical) and PLX-4720 (Symansis). The following antibodies were used in this study: AKT (Cell Signaling Technologies, 2920), pAKT (Cell Signaling Technologies, 9271), LRP6 (Cell Signaling Technologies, 3395), pLRP6 (Cell Signaling Technologies, 2568), ERK (Cell Signaling Technologies, 4696), pERK (Cell Signaling Technologies, 9101), CTNNB1 (Cell Signaling Technologies, 2698), pCTNNB1 (Cell Signaling Technologies, 9561), PARP1 (Cell Signaling Technologies, 9546), PKN1 (Santa Cruz Biotechnology, sc-136037), PKN2 (Bethyl Laboratories, A302-443A), pPKN (Cell Signaling Technologies, 2611), HA (Roche Applied Science, 3F10), GFP (Abcam, ab290), HSP90 (Abcam, ab13494), β-tubulin (Sigma-Aldrich, T7816), and AnnexinV (eBioscience, 88-8007-72).

##### Cell Lines, Western Blotting, Quantitative PCR, and Reporter Assays

The human melanoma cell lines A375, A2058, and Mel-624 were a generous gift from Cassian Yee (Fred Hutchinson Cancer Research Institute, Seattle, WA). The human melanoma cell lines COLO-829 and SKMEL5 were purchased from ATCC (Manassas, VA). Reporter cell lines were generated and assayed as described previously (27). Cell lines stably overexpressing FZD5, FZD7, LRP6, PKN1, or empty vector control were generated by retroviral transduction and puromycin selection. The cell lines L, A375, A2058, COLO-829, Mel-624, and SKMEL5 were cultured as described previously (17, 30). RNA isolation, quantitative PCR, protein lysis, fluorescence-activated cell sorting, and Western blotting were performed as described previously (17, 30, 31).

##### Surface Expression and Endocytosis Assays

Surface expression and internalization assays were carried out using an adaptation of a method described previously (32). HEK293T cells stably overexpressing HA-FZD5 and GFP-LRP6 were expanded onto 10-cm plates coated with poly-l-lysine (Sigma-Aldrich, P4707). 24 h after plating, cells were washed twice with cold supplemented PBS (PBS, 1 mm CaCl_2_, 0.5 mm MgCl_2_) and incubated with non-cell-permeable EZ-Link sulfo-NHS-SS-biotin (Thermo Scientific, 21331) at 1 mg/ml in PBS for 30 min at 4 °C. Following biotin labeling, cells were washed and then stimulated for 1 h at 4 °C. To allow internalization of surface molecules, the cells were then incubated for 30 min at 37 °C. Finally, the cells were washed twice for 15 min in cleavage buffer (75 mm NaCl, 10 mm EDTA) with tris(2-carboxyethyl)phosphine hydrochloride (Thermo Scientific, 20490) and processed for affinity chromatography and Western blotting.

## RESULTS

### 

#### 

##### The WNT3A-regulated Phosphoproteome in A375 Melanoma Cells

**FIGURE 1.**
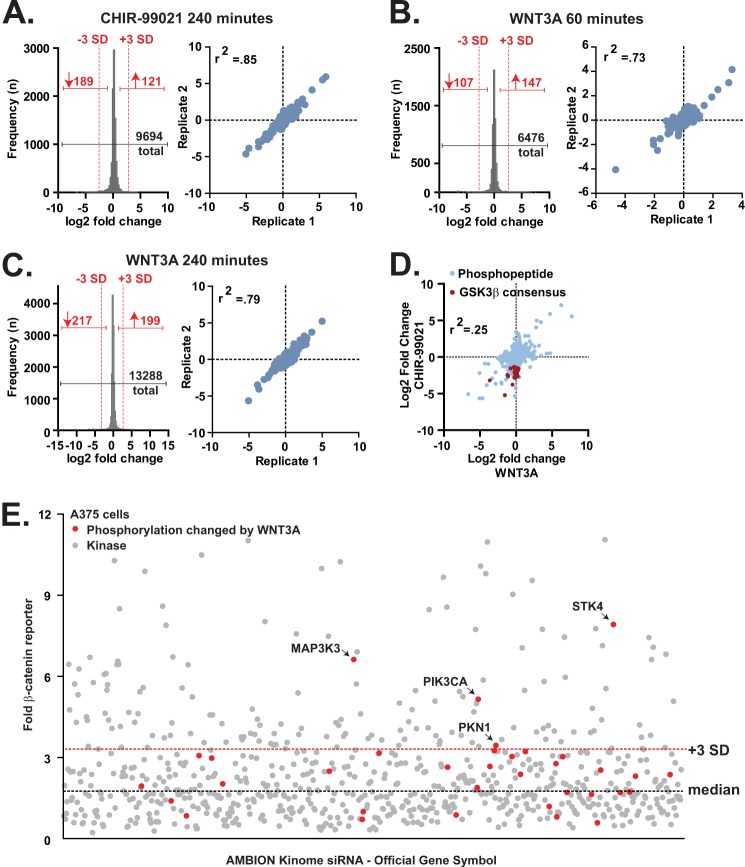
**Phosphoproteomics and siRNA screening identify PKN1 as a candidate kinase involved in Wnt/β-catenin signaling.** *A–C*, histograms (*left panel*) and scatter plots (*right panel*) showing the distribution of the log_2_ -fold change over vehicle treatment for phosphopeptides quantified from replicate CHIR-99021-stimulated (5 μm for 240 min; *A*) and WNT3A-stimulated (100 ng/ml for 60 min (*B*) and 100 ng/ml for 240 min (*C*)) samples. The *red dotted lines* represent the -fold change value corresponding to −3.0 and +3.0 population standard deviations from the median value of all peptides in the experiment. *D*, scatter plot showing the fold change values of phosphopeptides that were quantified from cells treated with CHIR-99021 and with WNT3A (60 min). Phosphopeptides containing the GSK3 consensus sequence Sp*XXX*Sp that decreased in abundance (<−3.0 population standard deviations from the median) following treatment with CHIR-99021 are highlighted with a *red* color. *E*, scatter plot of a kinome-based siRNA screen in A375 melanoma cells stably expressing BAR with each *dot* representing a known or predicted kinase. *Red dots* highlight proteins that contain phosphorylation sites that are altered in abundance following WNT3A stimulation (>3.0 or <−3.0 population standard deviations from the median). The *red dotted line* represents the -fold change equal to 3 population standard deviations greater than the median (*black dotted line*).

To better understand the functions of the Wnt/β-catenin signaling pathway in melanoma, we initially asked whether we could identify a subset of the phosphoproteome that is regulated by Wnt/β-catenin signaling. Specifically, we used stable isotope labeling by amino acids in cell culture (16) coupled with phosphopeptide enrichment (18). We stimulated duplicate cultures of A375 human melanoma cells with WNT3A for two periods of time to activate β-catenin signaling at the level of the receptors. We also activated β-catenin signaling downstream of the Wnt receptors using an orthogonal method: treatment of cells with CHIR-99021, a small molecule inhibitor of GSK3. We then quantified the relative abundance of each identified phosphopeptide relative to vehicle-treated controls. In total, we identified 19,601 unique phosphopeptides corresponding to 3396 distinct proteins (false discovery rate, 2.5%; Fig. 1, *A–C*, *left panel* and supplemental Databases S1–S3). We found the relative quantification of phosphopeptides in all three experiments to be highly reproducible between the duplicates (Fig. 1, *A–C*, *right panel*; CHIR-99021, Pearson *r*^2^ = 0.85; WNT3A 60 min, Pearson *r*^2^ = 0.73; and WNT3A 240 min, Pearson *r*^2^ = 0.79).

Although treating cells with either WNT3A ligand or with GSK3 inhibitor activates Wnt/β-catenin signaling, a comparison of the phosphoproteome regulated by WNT3A with that regulated by CHIR-99021 revealed significant differences. As expected, we found that peptides containing the GSK3 consensus sequence Sp*XXX*Sp (where Sp is phosphoserine) were largely decreased in abundance following treatment with CHIR-99021 (Fig. 1*D*, *red dots*), indicating their dephosphorylation following GSK3 inhibition. In contrast, most of these peptides did not change in abundance in WNT3A-stimulated A375 cells (Fig. 1*D*, *red dots*), suggesting that WNT3A does not broadly inhibit the kinase activity of GSK3 in melanoma cells.

##### siRNAs Targeting a Subset of the WNT3A-regulated Phosphoproteome Enhance Wnt/β-Catenin Signaling in Melanoma Cells

We next asked whether the WNT3A-regulated phosphoproteome might contain kinases that inhibit Wnt/β-catenin signal transduction. We subjected A375 malignant melanoma cells that harbor a luciferase reporter of β-catenin-dependent transcription (BAR) (27) to a 20% effective concentration (ED_20_) of WNT3A following transfection with siRNA pools targeting the 712 genes encoding known and predicted members of the human kinome. siRNAs targeting 179 kinases increased luciferase activity promoted by WNT3A stimulation (>3 standard deviations over universal control siRNA; Fig. 1*E* and supplemental Database S4). Of the kinases that we found to inhibit Wnt/β-catenin signaling, we observed that following WNT3A stimulation phosphopeptides corresponding to MAP3K3 (Ser-234 and Ser-237) and PIK3CA (Ser-231) increased in abundance and those corresponding to PKN1 (Ser-533) and STK4 (Thr-52) decreased in abundance (Fig. 1*E*, *red dots* and Table 1).

**TABLE 1:** **Wnt/β-catenin inhibitors that are regulated by WNT3A** For each of the listed gene symbols, the peptide sequences and fold changes in phosphopeptide abundance that were observed in the quantitative phosphoproteomics experiments are listed. Phosphorylation modifications are denoted with asterisks. A dash (—) indicates experiments where the listed peptide was not identified. Additionally, the -fold changes observed for the listed gene symbols in the siRNA screen are given. WNT3A-60, WNT3A stimulation for 60 min; WNT3A-240, WNT3A stimulation for 240 min.

Gene symbol	Peptide	log_2_ fold		
		WNT3A-60	WNT3A-240	siRNA screen
*MAP3K3*	LRS*ADS*ENALSVQER	4.72	−0.29	6.63
*PIK3CA*	S*MLLSSEQLK	—	3.89	5.16
*PKN1*	LIPNATGTGTFS*PGAS*PGSEAR	−2.84	−0.07	3.45
*STK4*	AIHKET*GQIVAIK	—	−3.47	7.92

##### Loss of PKN1 Synergizes with WNT3A to Elevate Wnt/β-Catenin Signaling

**FIGURE 2.**
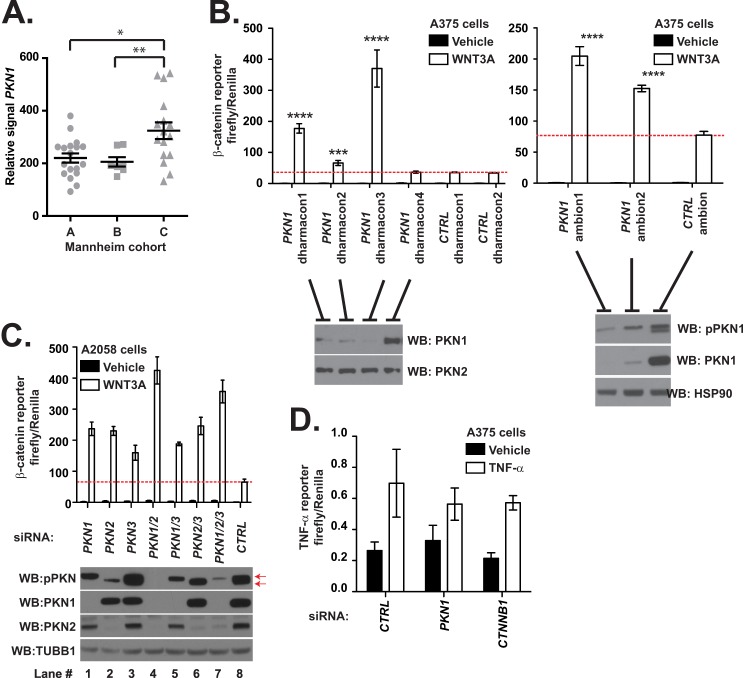
**Depletion of *PKN1* synergizes with WNT3A to elevate Wnt/β-catenin.** *A*, expression levels of PKN1 in the three Mannheim cohorts. These data were adapted from Hoek *et al.* (33). The *p* values (*, *p* < 0.05; **, *p* < 0.01) were calculated using a one-way ANOVA with Tukey's post-test. *B–D*, A375 (*B* and *D*) and A2058 (*C*) malignant melanoma cells stably expressing a β-catenin (*B* and *C*) or NF-κB (*D*) reporters were transiently transfected with the indicated siRNA oligonucleotides. Forty-eight hours after transfection, the cells were stimulated overnight with WNT3A-conditioned medium (*B* and *C*) or medium containing recombinant TNF (1 ng/ml; *D*) and then assayed for luciferase abundance. *Error bars* represent the S.D. of technical replicates, and the *p* value (****, *p* < 0.0001) was calculated using Student's *t* test. To assess the knockdown efficiency of the siRNAs, cells were lysed and processed for Western blot (*WB*) (*B* and *C*, *bottom panels*). *CTRL*, control.

The siRNA screening and high throughput phosphoproteomics analyses identified several protein kinases that regulate Wnt/β-catenin signaling in melanoma cells. To further assess whether these candidate kinases may be important in melanoma, we cross-referenced our data with a previously published and annotated malignant melanoma transcriptional profiling data set generated by Hoek *et al.* (33) (the Mannheim data set) that contains three distinct melanoma cohorts, one of which exhibits depressed Wnt/β-catenin signaling (cohort C). We found that compared with its expression in the A and B cohorts, PKN1 is significantly more abundant in cohort C (*p* < 0.01; Fig. 2*A* and Ref. 33). In contrast, a closely related kinase, PKN2, as well as the other candidate WNT3A-regulated phosphoproteins (STK4, PIK3CA, and MAP3K3), do not exhibit significant increases in abundance in cohort C.

Because elevated abundance of PKN1 is observed in a cohort of melanomas that exhibit decreased Wnt/β-catenin signaling (cohort C; Fig. 2*A*), we decided to further investigate its inhibitory effects on Wnt/β-catenin signaling in melanoma cells. To validate our siRNA screening findings, we treated A375 human melanoma cells with five distinct siRNAs targeting *PKN1* and then assayed the expression of the β-catenin-activated reporter in the presence or absence of exogenous WNT3A. This revealed that siRNAs that knock down *PKN1* (Fig. 2*B*) synergize with WNT3A to activate the Wnt/β-catenin reporter (Fig. 2*B*). These data indicate that depletion of *PKN1* can increase WNT3A-dependent reporter activity.

We observed that a commercial antibody that recognizes an activation loop phosphorylation of PKN1 (pPKN; Thr-774, a site distinct from that dephosphorylated following stimulation with WNT3A ligand) detects multiple bands in immunoblots of A375 (Fig. 2*B*, *right* blot) and A2058 (Fig. 2*C*, *lane 8*, see *arrows*) melanoma cell lines. One of these bands is depleted upon transfection with the *PKN1* siRNA (Fig. 2*C*, pPKN1 Western blot, *lane 1*, *lower* band), and another is depleted following transfection with siRNAs targeting the *PKN1* paralog, *PKN2* (Fig. 2*C*, pPKN1 Western blot, *lane 2*, *upper* band). To investigate whether PKN2, like PKN1, negatively regulates Wnt/β-catenin signaling in A2058 cells, we used siRNA transfection of oligos targeting *PKN1*, *PKN2*, or control sequences. We observed that cells transfected with siRNAs targeting *PKN2* exhibit increased WNT3A-dependent activity of the BAR in A2058 melanoma cells (Fig. 2*C*, *lane 2*). Additionally, we found that transfection of siRNAs targeting both *PKN1* and *PKN2* caused greater increases in WNT3A-dependent BAR activity than transfection of siRNAs targeting either gene in isolation (Fig. 2*C*, *lane 4*). Taken together, our data demonstrate that depletion of *PKN1* and *PKN2* can increase Wnt/β-catenin-dependent reporter activity.

##### Depletion of PKN Genes Increases the WNT3A-dependent Expression of AXIN2

**FIGURE 3.**
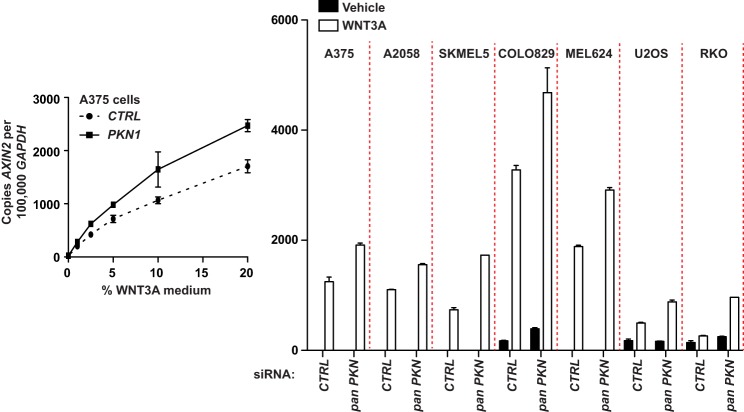
**PKN1 and PKN2 siRNAs increase expression of *AXIN2* in melanoma cell lines.** The indicated cell lines were transiently transfected with siRNA oligonucleotides targeting PKN1 and PKN2 (*pan PKN*) or targeting nonsense sequence (*CTRL*). Forty-eight hours after transfection the cells were stimulated overnight with medium conditioned with WNT3A or with vehicle (*left panel*, dose curve; *right panel*, single dose). The effects of WNT3A were assayed using quantitative PCR. cDNA was amplified using primers targeting *GAPDH* and the Wnt/β-catenin target gene *AXIN2*. The number of copies of *AXIN2* cDNA was averaged, normalized to 1 × 10^5^ copies of *GAPDH* amplified from the same sample, and plotted. The *error bars* represent S.D., and the data in all plots are representative of at least two independent experiments.

WNT3A-dependent BAR activity can be increased in response to many nonspecific perturbations. To verify that activation of the β-catenin reporter by *PKN1* siRNAs was not due to nonspecific elevation of luciferase activity, we first assessed the effects of siRNAs targeting *PKN1* on TNF-dependent activation of a luciferase reporter of NF-κB signaling (28). Following transfection of A375 melanoma cells with *PKN1* siRNAs, we found that TNF-dependent NF-κB signaling was not changed relative to siRNAs targeting control sequences (Fig. 2*D*). Next, to determine whether *PKN1* depletion regulates WNT3A-dependent transcriptional changes, we assessed the expression of the WNT3A target gene *AXIN2.* Following transfection of A375 malignant melanoma cells with siRNAs targeting *PKN1* or a control sequence and subsequent treatment with several doses of WNT3A, we used quantitative PCR to quantify the relative abundance of *AXIN2* relative to *GAPDH.* We found that the relative abundance of *AXIN2* was increased in the cells transfected with *PKN1* siRNAs at several doses of WNT3A (Fig. 3, *left panel*). These results support the idea that depletion of *PKN1* specifically increases WNT3A-dependent transcriptional responses.

Melanoma cells from different patients exhibit genetic heterogeneity that is not observed in any single cell line (34). To determine whether *PKN1* and *PKN2* inhibit Wnt/β-catenin-dependent gene expression in several established melanoma cell lines, we transfected siRNAs targeting *PKN1* and *PKN2* into A2058, SKMEL5, COLO-829, and Mel-624 (Fig. 3, *right panel*) cells. After WNT3A stimulation, we found that *AXIN2* transcripts increased in abundance in cells transfected with *PKN* siRNAs in all melanoma cell lines tested (Fig. 3, *right panel*). We then investigated the effects of *PKN1* and *PKN2* siRNAs on WNT3A-dependent *AXIN2* expression in osteosarcoma (U2OS), and colorectal carcinoma (RKO) cell lines (Fig. 3, *right panel*). We found that depletion of *PKN1* and *PKN2* caused increases in WNT3A-dependent accumulation of *AXIN2* transcripts in each of these cell lines, indicating that *PKN1* inhibits Wnt/β-catenin dependent transcription in a broad range of cell types.

##### Depletion of PKN1 Increases Apoptosis Initiated by WNT3A Stimulation

**FIGURE 4.**
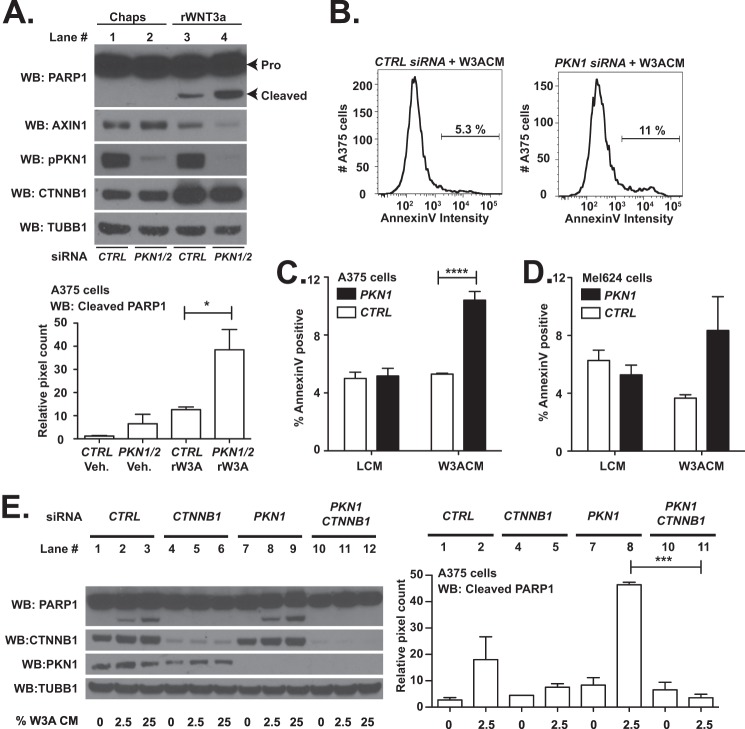
**Depletion of *PKN1* increases apoptosis initiated by WNT3A stimulation.** *A–E*, A375 and Mel-624 malignant melanoma cells were transfected with the indicated siRNA oligonucleotides. 48 h later, the cells were treated with medium containing recombinant WNT3A (*rWNT3A*; 100 ng/ml) or conditioned with WNT3A-secreting (*W3ACM*) or vehicle-secreting (*LCM*) L cells. To determine the effects of *PKN* gene depletion on melanoma cell death, Western blot (*WB*; *A* and *E*) or flow cytometry (*B–D*) analysis was performed. The abundance of cleaved PARP was used to assay for apoptosis in Western blot studies (*A*, *top panel*; *E*, left panel). Blots from three independent experiments were quantified by densitometry (*A*, *bottom panel*; *E*, *right panel*). As a second assay for apoptosis, the percentage of A375 (*B* and *C*) and Mel-624 (*D*) cells expressing AnnexinV was calculated. Representative plots (*B*) and quantification of three independent experiments (*C* and *D*) are presented. In *A*, *C*, *D*, and *E*, *error bars* represent S.E., and *p* values (*, *p* < 0.05; ***, *p* < 0.001; ****, *p* < 0.0001) were calculated using two-way ANOVA followed by a Bonferroni post-test. *CTRL*, control.

Previously, we have shown that melanoma cells undergo programmed cell death following continuous treatment with WNT3A (30). Because reducing expression of *PKN*1 and *PKN2* elevates Wnt/β-catenin-dependent transcription (Fig. 3), we predicted that *PKN* depletion would promote WNT3A-dependent melanoma cell apoptosis. To test this hypothesis, we transfected A375 melanoma cells with independent siRNAs directed against *PKN1* or against control sequences. Upon depletion of *PKN1*, we observed an increase in the abundance of WNT3A-induced cleaved PARP1 (Fig. 4*A*, compare *lane 4* with *lane 3*; quantification in *bottom panel*). To further address the role of *PKN* genes in melanoma apoptosis, we transfected A375 and Mel-624 malignant melanoma cell lines with *PKN1* and negative control siRNAs. Upon stimulation of the cells with WNT3A, we assessed the percentage of cells undergoing apoptosis using AnnexinV staining by flow cytometry. We observed increases in the percentage of cells expressing AnnexinV in A375 (representative images in Fig. 4*B* and quantification in Fig. 4*C*; *p* < 0.0001) and Mel-624 melanoma cells (Fig. 4*D*). We conclude that depletion of *PKN1* increases WNT3A-dependent cell death in A375 and Mel-624 melanoma cells.

To test whether *CTNNB1* is required for the increased apoptosis in melanoma cells that we observed upon depleting *PKN1*, we transfected A375 malignant melanoma cells with siRNAs targeting *PKN1* and *CTNNB1* individually and in combination. In contrast to A375 cells transfected with siRNAs targeting *PKN1* and a negative control sequence, we found that cells transfected with siRNAs targeting both *PKN1* and *CTNNB1* did not express detectable cleaved PARP1 upon stimulation with WNT3A (Fig. 4*E*, compare *lane 11* with *lane 8*; quantification in *right panel*). Thus, our results demonstrate that depletion of *PKN1* can synergize with WNT3A stimulation to promote apoptosis in malignant melanoma cells, and this effect requires *CTNNB1*.

##### Depletion of PKN1 Increases Apoptosis Initiated by a BRAF-V600E Inhibitor

**FIGURE 5.**
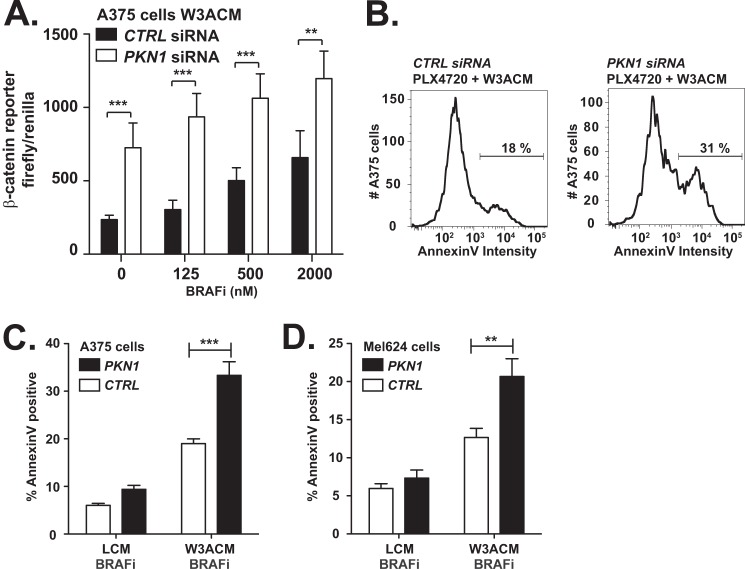
**Depletion of PKN1 increases apoptosis initiated by a BRAF-V600E inhibitor.** A375 and Mel-624 malignant melanoma cells were transfected with the indicated siRNA oligonucleotides. 48 h later, the cells were treated with BRAFi (2 μm PLX-4720 or as indicated) and medium conditioned with WNT3A-secreting (*W3ACM*) or vehicle-secreting (*LCM*) L cells. *A*, reporter activity was assayed in A375 malignant melanoma cells stably expressing a β-catenin reporter driving expression of firefly luciferase and a constitutive promoter driving *Renilla* luciferase. Flow cytometry was used to assay the percentage of A375 (*B* and *C*) and Mel-624 (*D*) cells expressing AnnexinV. Representative plots (*B*) and quantification of three independent experiments (*C* and *D*) are presented. *Error bars* represent the S.D. from at least three independent replicates, and the *p* values (**, *p* < 0.01; ***, *p* < 0.001) were calculated using a two-way ANOVA followed by a Bonferroni post-test. *CTRL*, control.

We have shown previously that co-treatment of several melanoma cell lines with WNT3A and with a targeted BRAF-V600E inhibitor synergistically increases Wnt/β-catenin signaling and apoptosis (30). To determine whether depletion of *PKN1* can further activate β-catenin signaling elicited by WNT3A and BRAF inhibitor (BRAFi) in melanoma, we transfected A375 cells harboring BAR with siRNAs targeting *PKN1* or control duplexes. Upon treatment with WNT3A, we observed increases in luciferase activity at all doses of the BRAFi PLX-4720. In cells transfected with *PKN1* siRNAs (Fig. 5*A*), we observed further increases in the luciferase reporter of β-catenin activity. To test whether depletion of *PKN1* also increases the number of cells undergoing programmed cell death upon treatment with BRAFi, we used flow cytometry to quantify the percentage of cells expressing the apoptosis marker AnnexinV (representative images in Fig. 5*B*). Consistent with our previous results (30), we observed that treatment of A375 (Fig. 5*C*) and Mel-624 (Fig. 5*D*) cells with WNT3A and BRAFi increased the percentage of cells expressing AnnexinV relative to cells treated with BRAFi alone. We further found that the percentage of melanoma cells expressing AnnexinV following stimulation with WNT3A and BRAFi was significantly increased in cells transfected with siRNAs targeting *PKN1* as compared with those transfected with negative control siRNA (A375 representative plots in Fig. 5*B* and quantification in Fig. 5*C*, *p* < 0.01; Mel-624 quantification in Fig. 5*D*, *p* < 0.001). These data suggest that inhibiting PKN1 may increase melanoma sensitivity to BRAFi in the context of active Wnt/β-catenin signaling.

##### PKN1 Forms a Complex with the WNT3A Receptor Frizzled7

**FIGURE 6.**
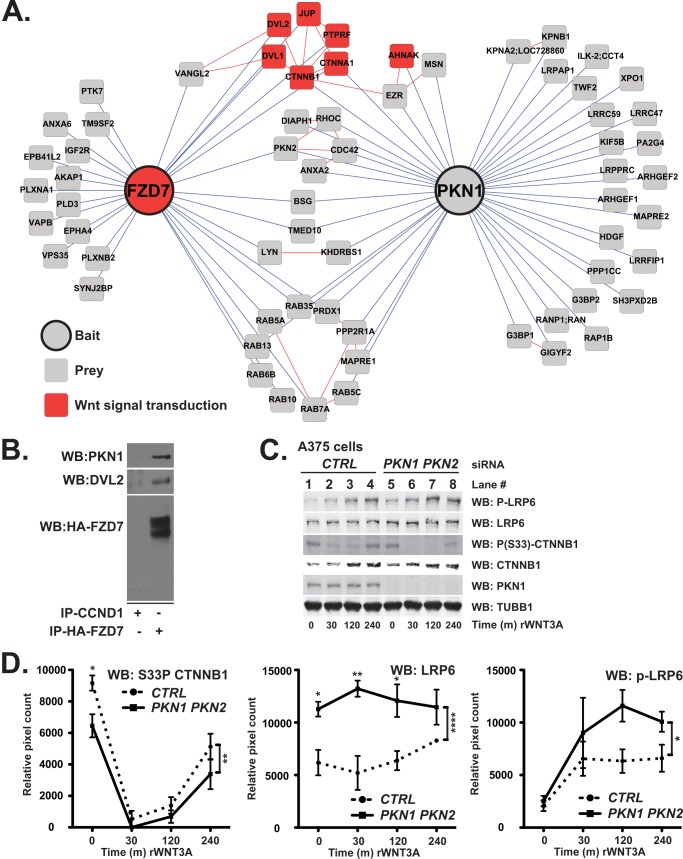
**PKN1 co-purifies with the WNT3A receptor FZD7, and its depletion promotes phosphorylation changes associated with Wnt/β-catenin signaling.** *A*, proteins (nodes) are represented as *circles* (bait) or *squares* (prey) with the relationships between nodes (edges) denoted by the connecting *lines*. A protein-protein interaction network for FZD7 and PKN1 was generated from primary affinity purification-mass spectrometry data (*blue* edges) and from literature-curated interactions (*red* edges). *Red* nodes represent bait or prey that has been previously reported to regulate Wnt/β-catenin signaling. *B*, HA or the negative control CCND1 was immunoprecipitated (*IP*) from lysates isolated from HEK293T cells stably expressing HA-FZD7 and analyzed by Western blot (*WB*). *C* and *D*, A375 malignant melanoma cells were transiently transfected with the indicated siRNA oligonucleotides. 62 h after transfection, the cells were stimulated for the indicated times and analyzed by Western blot with the indicated antibodies. *D*, Western blots from three independent experiments were quantified by densitometry for phosphorylated CTNNB1 (*P(S33)CTNNB1*; *left panel*), total LRP6 (*LRP6*; *middle panel*), and phosphorylated LRP6 (*p-LRP6*; *right panel*). *Error bars* represent S.E., and *p* values (*, *p* < 0.05; **, *p* < 0.01; ****, *p* < 0.0001) were calculated using two-way ANOVA (*right brackets*) followed by a Bonferroni post-test (individual time points). *CTRL*, control.

Given that there have heretofore been no reports of PKN1 modulating Wnt/β-catenin signaling, we next initiated experiments to determine whether PKN1 can be detected in complexes containing known components of the Wnt/β-catenin pathway. We therefore screened for binding partners of PKN1 using affinity purification and mass spectrometry in A375 melanoma cells (supplemental Database S5). This approach identified PKN1 as co-purifying with membrane-associated proteins known to regulate Wnt/β-catenin signal transduction (Fig. 6*A*). In addition, we detected several proteins known to regulate receptor-mediated endocytosis co-purifying with PKN1 (Fig. 6*A*). In parallel experiments, we used affinity purification-mass spectrometry to identify binding partners of the WNT3A receptor FZD7 (supplemental Database S6). We were surprised to find that an overlapping set of proteins that includes PKN2 co-purify with PKN1 and FZD7 (Fig. 6*A*). Moreover, we found that epitope-tagged FZD7 co-purified with PKN1 and DVL2 in HEK293T cells (Fig. 6*B*), further suggesting that a pool of PKN1 resides within a protein complex including FZD7.

Based on our data that a pool of PKN1 is in complex with FZD7, we predicted that depletion of PKN proteins should alter WNT3A-dependent biochemical events immediately downstream of receptor activation. Following treatment of A375 melanoma cells with WNT3A, phosphorylation sites on CTNNB1 typically regulated by GSK3 become dephosphorylated, and as CTNNB1 accumulates, those sites are again phosphorylated (Fig. 6*C*, time course in *lanes 1–4*). We found that siRNAs targeting the PKN proteins significantly decrease the abundance of the GSK3-phosphorylated form of CTNNB1 in A375 (Fig. 6*C*, compare expression of *P(S33)-CTNNB1* in *lanes 5–8* with that in *lanes 1–4*; quantification is in Fig. 6*D*, *left panel*) melanoma cells following stimulation with WNT3A. These data indicate that PKN1 inhibits the Wnt/β-catenin pathway upstream of GSK3.

In addition to dephosphorylation of CTNNB1, WNT3A stimulation results in increased abundance of the phosphorylated form of LRP6, a WNT3A co-receptor (Fig. 6*C*, compare expression of *P-LRP6* in *lanes 5–8* with that in *lanes 1–4*; quantification is in Fig. 6*D*, *right panel*). Additionally, we have consistently observed a higher abundance of total LRP6 in A375 melanoma cells transfected with siRNAs targeting the PKN proteins relative to that in cells transfected with negative control siRNAs (Fig. 6*C*; quantification is in Fig. 5*D*, *middle panel*). Taken together, our biochemical findings support the hypothesis that PKN1 and PKN2 inhibit Wnt/β-catenin signaling at the level of the plasma membrane.

##### Inhibition of PKN1 Increases Surface Expression of LRP6

**FIGURE 7.**
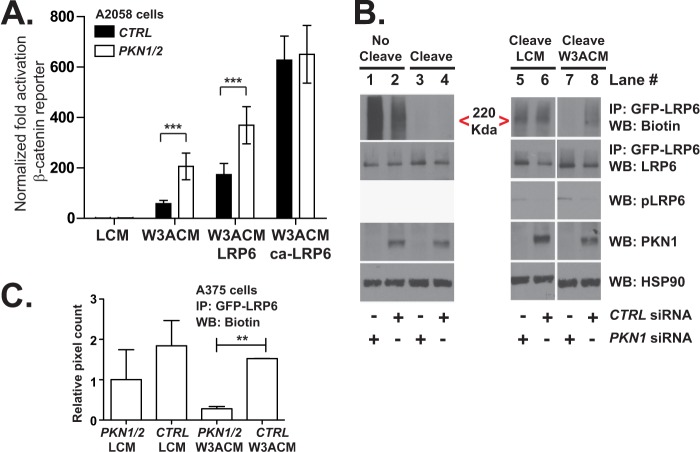
**PKN1 may attenuate Wnt/β-catenin signaling by inhibiting internalization of LRP6.** *A*, 48 h following transfection of the siRNAs, A2058 cells were transiently transfected with plasmids expressing BAR, a constitutive promoter driving *Renilla* luciferase, and either empty vector, wild type LRP6, or truncated LRP6. The next day, luciferase abundance was assayed and plotted. The *error bars* represent S.D. from four replicates. *B* and *C*, HEK293T cells stably overexpressing GFP-LRP6 were transiently transfected with siRNA oligonucleotides targeting the indicated genes. 62 h later, the cells were serum-starved on ice and biotinylated with sulfo-NHS-SS-biotin. Following labeling, cells were stimulated and subsequently brought to 37 °C to initiate internalization of the biotinylated receptors. To cleave the remaining surface biotin, the samples were incubated with the reducing agent tris(2-carboxyethyl)phosphine. Lastly, cells were lysed, processed for immunoprecipitation (*IP*) using a GFP antibody, and analyzed by Western blot (*WB*). *C*, Western blots for biotin present in the GFP-LRP6 immunoprecipitation from two independent experiments were quantified by densitometry and plotted. *Error bars* represent S.E., and the *p* values (**, *p* < 0.01) were calculated using one-way ANOVA followed by Tukey's post test. In *A*, the *p* values (***, *p* < 0.001) were calculated using two-way ANOVA followed by a Bonferroni post-test. *CTRL*, control; *W3ACM*, WNT3A-conditioned medium; *LCM,* L cell-conditioned media; *ca-LRP6*, constitutively active LRP6.

Our biochemical data suggest that PKN1 co-purifies with several proteins known to regulate endocytic trafficking and that depletion of *PKN1* alters the expression and phosphorylation of LRP6. LRP6 is internalized upon stimulation with WNT3A. To determine whether *PKN1* depletion can alter LRP6-dependent signaling after it is internalized, we used isoforms of LRP6 that lack the extracellular domain that are constitutively internalized (35) and are strong activators of the Wnt/β-catenin pathway. We found that siRNAs targeting *PKN1* and *PKN2* enhance BAR activity induced by full-length, but not truncated, LRP6 (Fig. 7*A*). These data suggest that PKN proteins regulate LRP6 upstream of its internalization.

One explanation for our data is that PKN1 regulates internalization of LRP6. To monitor the cellular internalization of WNT3A receptors, we labeled HEK293T cells stably transfected with GFP-tagged versions of LRP6 with non-membrane-permeable biotin. To assess whether the surface expression of GFP-LRP6 is altered by *PKN1* siRNAs, we determined the relative amounts of biotin present in GFP immunoprecipitates. We found that biotinylated GFP-LRP6 runs as a high molecular mass smear around 220 kDa (Fig. 7*B*, see *lane 1* next to the *caret*) due to the variable number of biotin molecules that are attached to the protein. In cells transfected with *PKN1* siRNAs, we observed that the abundance of biotinylated LRP6 (Fig. 7*B*, compare bands next to the *red caret* in *lane 2* with those in *lane 1*) was increased relative to cells transfected with control siRNAs, indicating that depletion of PKN1 increases the surface expression of LRP6. To determine whether the observed increases in surface LRP6 in *PKN1*-depleted cells were caused by decreased internalization of LRP6, we quantified internalized biotin. To do so, we cleaved the surface biotin and affinity-purified LRP6-GFP. We found that the abundance of biotinylated GFP-LRP6 in the cleaved samples was significantly decreased in HEK293T cells transfected with siRNA targeting *PKN1* relative to that in cells transfected with negative control siRNA (Fig. 7*B*, compare bands next to the *red caret* in *lanes 8* with those in *lane 7*; quantification shown in Fig. 7*C*). Taken together, our biochemical data show that PKN1 associates with membrane proteins and that it promotes internalization of LRP6, which is likely to contribute to its inhibition of Wnt/β-catenin signaling.

## DISCUSSION

Data from high throughput siRNA screens (17, 22, 36) and affinity purification-mass spectrometry experiments (17, 22, 29, 31, 37) reveal complex regulation of the Wnt/β-catenin pathway by kinases and phosphatases. One observation that we made in this study is that phosphorylation changes catalyzed by stimulation of melanoma cells with the WNT3A ligand are distinct from those caused by inhibition of GSK3. We found that the subset of phosphopeptides that are decreased in abundance following GSK3 inhibition that contain a GSK3 consensus site (Sp*XXX*Sp) are largely unaffected by stimulus with the WNT3A ligand. These findings contrast with the model presented by Taelman *et al.* (38) that proposes that the WNT3A ligand promotes trafficking of the majority of GSK3 to multivesicular bodies, thus sequestering GSK3 and preventing it from regulating cytosolic proteins. Instead, our data suggest that the WNT3A stimulus does not generally regulate proteins phosphorylated by GSK3 and are consistent with earlier studies that demonstrate that Wnt ligands can inhibit the ability of GSK3 proteins to phosphorylate CTNNB1 but not necessarily other substrates (39). One possible explanation for the contrast with the earlier study is that the majority of GSK3 substrates may migrate with and remain accessible to GSK3 in multivesicular bodies upon cellular stimulation with WNT3A. Regardless, our data suggest that inhibiting GSK3 is not synonymous with activating the Wnt/β-catenin pathway, which is an important consideration when using GSK3 inhibition as a proxy for activation of β-catenin by Wnt ligands.

We found that WNT3A promotes phosphorylation changes in several proteins that have been previously reported to regulate RAS activation and phosphatidylinositol 3-kinase signaling (MTOR, RICTOR, PIK3CA, and RPS6KA4). Previous work has demonstrated that forced expression of a non-degradable phosphorylation mutant of CTNNB1 can increase the metastatic potential of melanoma cells harboring activating mutations in the NRAS/phosphatidylinositol 3-kinase pathways (40, 41). Because many of the WNT3A-dependent changes in phosphorylation that we observed occurred within 60 min of stimulus, our data suggest that the WNT3A ligand may also regulate progrowth pathways (*e.g.* mitogen-activated protein kinase and phosphatidylinositol 3-kinase signaling) in melanoma cells that are possibly independent of CTNNB1.

An interesting finding in these studies is that *PKN1* depletion increases LRP6 cell surface expression and phosphorylation at serine 1490, a site associated with active Wnt/β-catenin signaling. Similarly, additional studies have shown that manipulations that increase surface expression of FZD (42, 43) or LRP6 (44, 45) also increase expression of downstream markers of Wnt/β-catenin signaling. Collectively, these findings are inconsistent with the hypothesis that internalization of LRP6 is necessary for downstream Wnt/β-catenin signal transduction.

Two models have been proposed to explain how Wnt ligands inhibit GSK3, enabling stabilization of CTNNB1 (46). The first is based on the idea that the WNT3A stimulus causes recruitment of GSK3 to the cell membrane where it is subsequently internalized into multivesicular bodies (38), thus allowing cytosolic CTNNB1 to accumulate. In contrast, a second model proposes that phosphorylated LRP6 binds GSK3 and inhibits its catalytic activity (47–50). Our data showing that PKN1 depletion increases LRP6 internalization while simultaneously decreasing GSK3-dependent phosphorylation of CTNNB1 are more consistent with the latter model.

PKN1 is an atypical PKC kinase that acts as a Rho effector in several contexts and can regulate AKT (51–54) and MAPK8 (also known as Jnk) (55) signaling (for a review, see Ref. 56). PKN proteins localize to the endoplasmic reticulum and the endosome (57–59) and are known to regulate vesicular traffic (60). A protein array-based screen for substrates of the PKN1 kinase determined that it can phosphorylate several receptor proteins including EPHA5, EGF receptor, RET, and GRK4 (61). Our finding that PKN1 is required for WNT3A-dependent internalization of LRP6 is consistent with data showing that PKN1 promotes RHOB-dependent endocytosis of the EGF receptor (58). Also consistent with these findings, we observed that PKN1 co-purifies with several proteins known to regulate vesicle trafficking. Based on these findings, we hypothesize that PKN proteins might directly regulate surface expression of several receptor proteins including LRP6. However, because PKN1 is known to regulate the AKT (54) and mitogen-activated protein kinase pathways (55, 62, 63), its effects on WNT3A-dependent signaling could be mediated indirectly via its effects on those pathways. Further research to clarify how PKN1 regulates receptor dynamics may greatly enhance our understanding of the mechanisms governing LRP6 cell surface expression.

Finally, our data showing that depletion of PKN1 increases WNT3A-dependent apoptosis in melanoma cells bolster previous data that PKN1 may be relevant to cancer biology: 1) the depletion of PKN1 also promotes programmed cell death in models of multiple myeloma (64), 2) PKN1 is overexpressed in prostate tumors (65) and in certain cohorts of malignant melanoma (Fig. 2*B*), and 3) PKN1 is a downstream effector of PDPK1, which is activated during phosphatidylinositol 3-kinase signaling (59). Finally, our demonstration that depletion of *PKN1* increases the number of cells undergoing programmed cell death upon treatment with BRAFi could contribute to improvements in therapies.

## Footnotes

5 The abbreviations used are:

FZD: Frizzled
LRP: low density lipoprotein receptor-related protein
BAR: β-catenin-activated reporter
PKN1: protein kinase N1
pPKN1: phosphorylated protein kinase N1
BRAF: v-*raf* murine sarcoma viral oncogene homolog B
BRAFi: BRAF inhibitor
GSK3: glycogen synthase kinase 3
EB: elution buffer
ANOVA: analysis of variance
PARP: poly(ADP-ribose) polymerase.
